# Novel insight into the genetic diversity of strongylid nematodes infecting South-East and East Asian primates

**DOI:** 10.1017/S0031182024000386

**Published:** 2024-04

**Authors:** Bethan Mason, Barbora Cervena, Liesbeth Frias, Benoit Goossens, Hideo Hasegawa, Kenneth Keuk, Abdullah Langgeng, Kasia Majewski, Takashi Matsumoto, Keiko Matsuura, Renata Mendonça, Munehiro Okamoto, Steve Peter, Klara J. Petrzelkova, Symphorosa Sipangkui, Zhihong Xu, Barbora Pafco, Andrew J.J. MacIntosh

**Affiliations:** 1 Department of Botany and Zoology, Faculty of Science, Masaryk University, Brno, Czech Republic; 2 Institute of Vertebrate Biology, Czech Academy of Sciences, Brno, Czech Republic; 3 Programme in Emerging Infectious Diseases, Duke-NUS Medical School, Singapore; 4 Danau Girang Field Centre, c/o Sabah Wildlife Department, Kota Kinabalu, Sabah, Malaysia; 5 Organisms and Environment Division, Cardiff School of Biosciences, Cardiff University, Cardiff, UK; 6 Department of Biomedicine, Faculty of Medicine, Oita University, Oita, Japan; 7 Wildlife Research Center, Kyoto University, Inuyama Campus, Inuyama, Japan; 8 Department of Environmental and Preventive Medicine, Faculty of Medicine, Oita University, Japan; 9 Wildlife Research Center, Kyoto University, Kyoto, Japan; 10 Centre for Functional Ecology – Science for People & the Planet, Department of Life Sciences, University of Coimbra, Coimbra, Portugal; 11 Center for the Evolutionary Origins of Human Behavior (EHUB), Kyoto University, Kyoto, Japan; 12 Kulliyah of Science, Department of Biotechnology, International Islamic University of Malaysia, Kuantan, Pahang, Malaysia; 13 Institute of Parasitology, Biology Centre, Czech Academy of Sciences, České Budějovice, Czech Republic; 14 Liberec Zoo, Liberec, Czech Republic; 15 Sabah Wildlife Department, Kota Kinabalu, Sabah, Malaysia

**Keywords:** helminth, metabarcoding, non-human primate, orangutan, strongylida

## Abstract

With many non-human primates (NHPs) showing continued population decline, there is an ongoing need to better understand their ecology and conservation threats. One such threat is the risk of disease, with various bacterial, viral and parasitic infections previously reported to have damaging consequences for NHP hosts. Strongylid nematodes are one of the most commonly reported parasitic infections in NHPs. Current knowledge of NHP strongylid infections is restricted by their typical occurrence as mixed infections of multiple genera, which are indistinguishable through traditional microscopic approaches. Here, modern metagenomics approaches were applied for insight into the genetic diversity of strongylid infections in South-East and East Asian NHPs. We hypothesized that strongylid nematodes occur in mixed communities of multiple taxa, dominated by *Oesophagostomum*, matching previous findings using single-specimen genetics. Utilizing the Illumina MiSeq platform, ITS-2 strongylid metabarcoding was applied to 90 samples from various wild NHPs occurring in Malaysian Borneo and Japan. A clear dominance of *Oesophagostomum aculeatum* was found, with almost all sequences assigned to this species. This study suggests that strongylid communities of Asian NHPs may be less species-rich than those in African NHPs, where multi-genera communities are reported. Such knowledge contributes baseline data, assisting with ongoing monitoring of health threats to NHPs.

## Graphical abstract

**Figure:**
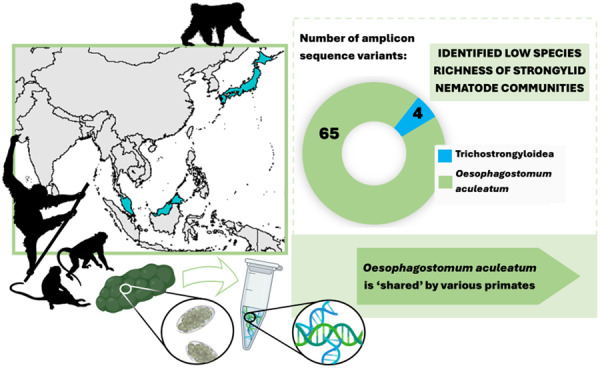

## Introduction

Non-human primates (NHPs) are among the most endangered of mammalian taxa (Estrada *et al*., 2017; Fernández *et al*., 2022). Asian NHPs are particularly threatened, with one of the largest proportions of threatened NHP species reported across global regions, second only to Madagascar (Fernández *et al*., 2022). Asian NHPs commonly feature among the International Union for Conservation of Nature's ‘World's 25 Most Endangered Primates’ and face a variety of conservation challenges (Mittermeier *et al*., 2006). Consequently, effective conservation strategies rely on understanding both their ecology and conservation threats. One such threat is the risk of disease, with various bacterial, viral and parasitic infections previously reported to have damaging consequences for NHP hosts (Chapman *et al*., 2005; Mul *et al*., 2007; Gillespie *et al*., 2008; Bicca-Marques *et al*., 2022). In particular, host–parasite interactions have long been associated with population dynamics of wildlife hosts (Hudson and Dobson, 1995). Wild NHPs host numerous parasites (Nunn *et al*., 2003; Frias and MacIntosh, 2020), including strongylid nematodes, a major radiation of parasitic helminths, herein referred to as strongylids. Strongylids are amongst the most common gastrointestinal parasites of wild NHPs, typically occurring as asymptomatic infections within such hosts (Cantacessi *et al*., 2012; Pafčo *et al*., 2018). However, clinical manifestations have been previously reported in both captive and wild NHPs (Pit *et al*., 2001; Mul *et al*., 2007; Muhangi *et al*., 2021). Strongylids are expected to occur in complex communities of multiple genera within NHP hosts, as is conventional in large terrestrial herbivores (Pafčo *et al*., 2018).

While strongylids have been widely studied in both human medicine and veterinary sciences (Zajac, 2006; Cantacessi *et al*., 2012), knowledge of these infections in wildlife is still limited (Krief *et al*., 2008; Mclean *et al*., 2012; Walker and Morgan, 2014). It is restricted by the indistinguishability of different species, or even genera, through traditional coproscopic approaches (Jex *et al*., 2011; Pafčo *et al*., 2018). Previously applied molecular approaches are time consuming and require either single larval specimens or species-specific primers. Such methodologies are insufficient for capturing the true genetic diversity of these complex communities, at risk of overlooking rarer taxa (Pafčo *et al*., 2018). Technological advancements in high-throughput sequencing (HTS) now facilitate simultaneous sequencing of mixed DNA from entire communities, including identification of rare taxa (von Bubnoff, 2008), in a timely and cost-effective manner. In recent research, HTS methodologies have confirmed the presence of complex strongylid communities in various African NHPs (Pafčo *et al*., 2019; Mason *et al*., 2022). However, application is not currently widespread and these methods are yet to be applied to strongylid communities of Asian NHPs.

Recent research has revealed striking differences in strongylid communities of African great apes (Pafčo *et al*., 2019; Ilík *et al*., 2023), raising questions as to the drivers of these complex communities. Parasite phylogenies are sometimes thought to emulate the host phylogenies within which they evolved, as predicted by the host-parasite co-speciation hypothesis (Brooks, 1979). However, environmental parameters, such as temperature, humidity or vegetation, are known to influence longevity and transmission of parasite populations (Silangwa and Todd, 1964; Callinan and Westcott, 1986; Jex *et al*., 2011). As soil-transmitted helminths, strongylids may be particularly influenced by environmental variation, due to development of infectious larvae involving free-living larval stages within the external environment (Kalousová *et al*., 2014; Knapp-Lawitzke *et al*., 2014). Similarly, parasite populations are also influenced by the presence or absence of sympatric species, be that other wildlife or domestic animals (Hatcher *et al*., 2012). Existence of multiple potential hosts within a given area facilitates parasite sharing between host species, particularly among phylogenetically related hosts (Dallas *et al*., 2019), including NHPs (Cooper *et al*., 2012; Pafčo *et al*., 2019). With a direct lifecycle, strongylids rely on overlapping host ranges for transmission (Pafčo *et al*., 2019), without an intermediate or transport host to facilitate wider transmission.

Coproscopic studies have confirmed a high prevalence of strongylids in both South-East and East Asian primates (Arizono *et al*., 2012; Frias *et al*., 2021). To date, genetic research of strongylids in Asian NHPs has typically focused on *Oesophagostomum* spp., the so-called ‘nodular worms’. Only one species of nodular worm, *O. aculeatum*, has been genetically identified in Asian NHPs thus far (Arizono *et al*., 2012; Frias *et al*., 2019; Yalcindag *et al*., 2021). There are few reports of other strongylid taxa in Asian NHPs. For example, a previous molecular characterization identified the presence of a single *Ternidens deminutus* larva in a sampled community of Bornean NHPs (Frias *et al*., 2019). However, due to this previous research using single larval specimens and relying on taxa-specific primers, the strongylid communities identified cannot be deemed conclusive. To build upon current knowledge, a holistic approach better able to capture the entire strongylid community, including rare taxa, is needed.

Here, a previously optimized HTS approach (Pafčo *et al*., 2018) is utilized to shed light on the genetic diversity of strongylid communities infecting South-East and East Asian NHPs. This approach is applied to 5 distinct NHP populations, including a total of 6 primate species. In describing the strongylid communities of Bornean orangutans (*Pongo p*y*gmaeus*), one of the only Asian great apes, and several sympatric NHP species, the aim is to provide a unique insight into the host–parasite co-speciation hypothesis within great apes. Insight into within species variation of NHP strongylid communities is also provided, through comparison of Japanese macaques (*Macaca fuscata)* from 3 isolated localities. We hypothesize that strongylid nematodes occur in mixed communities of multiple taxa, dominated by *Oesophagostomum*, matching previous findings based on single-specimen genetics.

## Material and methods

### Sample collection

In this study, 90 individual faecal samples were utilized, non-invasively collected from 6 different wild NHP species in South-East and East Asia, precisely across 5 localities in Malaysian Borneo and Japan. In Borneo, sampling was conducted at 2 localities: the Lower Kinabatangan Wildlife Sanctuary (LKWS) and Danum Valley Conservation Area (DVCA), both of which occur in the state of Sabah. At LKWS, 36 samples representing 5 primate species were collected: crab-eating macaque (*Macaca fascicularis*, *n* = 17), southern pig-tailed macaque (*Macaca nemestrina*, *n* = 2), proboscis monkey (*Nasalis larvatus*, *n* = 4), silvery lutung (*Trachypithecus* cristatus, *n* = 5), and Bornean orangutan (*Pongo pygmaeus*, *n* = 2). Except for Bornean orangutans, which were either followed or sampled opportunistically during their visits to the Centre, LKWS samples were collected during systematic boat surveys along the Kinabatangan River, with NHP species being opportunistically sampled when encountered at sleeping sites early in the morning. Visual appearance of faeces based on softness and presence of invertebrates was used to ensure that only fresh samples were collected. Finally, 12 identified Bornean orangutans inhabiting DVCA were sampled during standard focal follows of habituated individuals. Further sampling was conducted in Japan, with 48 samples collected from the 2 subspecies of Japanese macaques (*Macaca fuscata fuscata* and *M. f. yakui*) inhabiting 3 localities: Jigokudani (*n* = 15), Koshima (*n* = 21) and Yakushima (*n* = 12). At these 3 localities, the primates are habituated to human presence and each sample can be attributed to a known individual. Samples from all localities were fixed in ethanol as soon as possible after collection, always on the same day, with fixed samples then stored at environmental temperature before transportation to Wildlife Research Center, Kyoto University, Japan.

### DNA isolation and sequencing

Total genomic DNA was extracted from faecal samples using a QIAamp DNA stool mini kit (Qiagen, Japan) following the manufacturer's recommendations. To ensure correct host identification of samples originating from unidentified hosts in LKWS, cytochrome b (*cytb*) gene fragments were amplified using the primer pair L14724/H15915 (CGAAGCTTGATATGAAAAACCATCGTTG/AACTGCAGTCATCTCCGGTTTACAAGAC – respectively) (Irwin *et al*., 1991). Amplicons were then purified using the Agencourt AMPure system (Agencourt Bioscience Corp., USA) before sequencing in an ABI 3730xl DNA Analyzer (Applied Biosystems, USA). Resulting sequences were compared with GenBank template sequences to identify the correct primate species, described in full in Frias *et al*. (2018).

Strongylid presence was assessed in each sample using HTS, where the entire strongylid community was determined by amplification of the second internal transcribed spacer (ITS-2) of nuclear DNA through PCR (polymerase chain reaction) using the forward primer Strongyl_ITS-2_F (ACGTCTGGTTCAGGGTTG) and the reverse Strongyl_ITS-2_R (ATGCTTAAGTTCAGCGGGTA) (Pafčo *et al*., 2018). To generate HTS sequencing libraries a 2-step PCR approach that employs Nextera primer design was used (Pafčo *et al*., 2018), with each sample tagged with a unique primer barcode, following Illumina libraries guide. Through the Illumina MiSeq platform the final library was sequenced using MiSeq Reagent Kit version 2 (2 × 250-bp pair-end reads, 500 cycles).

### Bioinformatics and data analysis

Gene-specific primers were trimmed using SKEWER (Jiang *et al*., 2014). Using DADA2, paired end reads were assembled (Callahan *et al*., 2016). The dataset was then denoised, low-quality reads (expected error rate >1) eliminated and inflation of strongylid diversity by PCR/sequencing artefacts (chimeras) avoided by corrections in DADA2 (Callahan *et al*., 2016). To taxonomically assign amplicon sequence variants (ASVs) the naïve Bayesian RDP classifier (Wang *et al*., 2007) was employed in the DADA2 pipeline. With strongylid ITS-2 sequences, downloaded from the NCBI nr/nt database (200 top BLASTN hits for each ASVs), a training database of reference sequences for assignment was constructed. The dataset was then filtered to remove all unclassified ASVs, those not assigned to Strongylida. Phylogenetic maximum likelihood analysis was performed using the web version of IQ TREE (Trifinopoulos *et al*., 2016), with integrated ModelFinder selecting the most suitable model based on Bayesian information criterion (BIC) (Kalyaanamoorthy *et al*., 2017), after removal of outlying ASV sequences with both low abundance and prevalence and addition of *Oesophagostomum* spp. sequences available in the GenBank. The topology of phylogenetic trees was tested using 1000 replicates of ultrafast bootstrap (Hoang *et al*., 2018) and Shimodaira–Hasegawa approximate likelihood ratio test (SH-aLRT) (Anisimova *et al*., 2011). Through the model outputs, attention was also paid to pairwise distances between taxa, which were calculated from the alignments guided by Clustal Omega integrated into Geneious Prime 2023 2.1 (http://www.geneious.com). The trees were visualized and annotated in iTOL v5 (Letunic and Bork, 2021).

Differences in alpha diversity of strongylid communities were investigated by using the number of ASVs per sample as a proxy measure. Using the lme4 R package (de Boeck *et al*., 2011; Bates *et al*., 2015), a GLMM (generalized linear mixed model) with negative binomial distribution was applied, accounting for aggregated parasite distributions, to assess the effect of host species on alpha diversity, including study site as a random factor. To assess overdispersion and ensure correct model selection, residual diagnostics were implemented and visually inspected through the DHARMa R package (Hartig, 2020), with no significant deviation found. Then the drop1() function (again in lme4 R package) was implemented to determine the importance of host species as a model effector, with strong significance identified comparative to a null hypothesis. *Post hoc* comparisons, using Tukey Honest Significant Differences, were employed to test the effect of host species factorially. Using the vegan R package (Oksanen *et al*., 2019), between species variation in beta diversity, at the individual ASV level, was investigated through ANOSIM-based community compositional dissimilarities and tests of multivariate homogeneity, employing Bray–Curtis dissimilarities to account for ASV relative abundances. Clustering was visualized through weighted PCoA (principal coordinates analysis), again using the vegan R package (Oksanen *et al*., 2019). To investigate within species variation of beta diversity, the same methods were applied to a data subset including only Japanese macaques, employing host locality as the effector variable.

## Results

**Figure 1.**
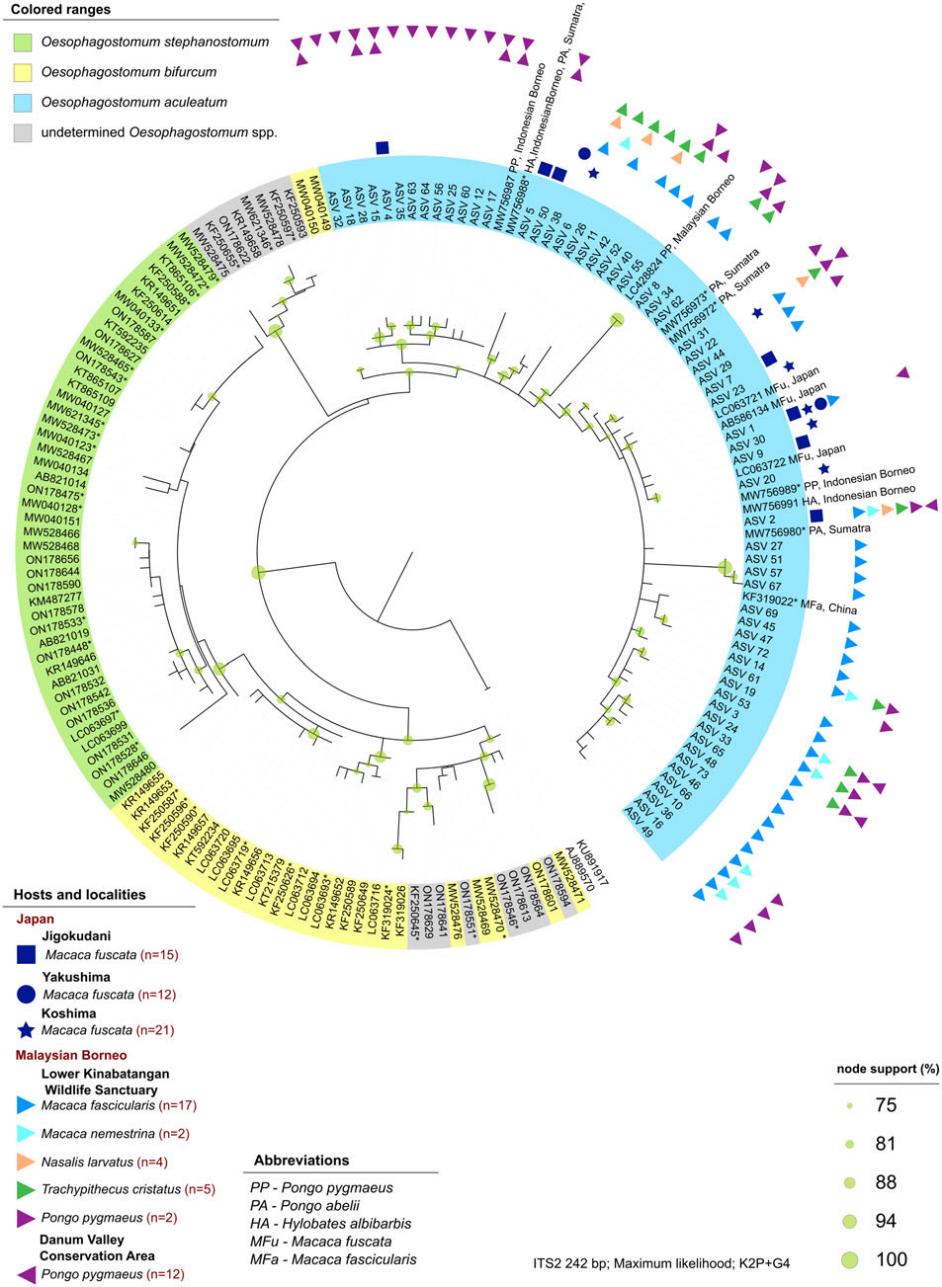
Maximum likelihood cladogram of *Oesophagostomum* ITS-2 region (245 bp), computed in IQ tree3 by model K2P + G4, using *O. dentatum* as an outgroup. The tree topology was tested by 1000 replicates of ultrafast bootstrap4 and SH-like aLRT5. Green circles mark nodes with support higher than 75%, with circle size depicting value. Sequences from GenBank are labelled by accession number.

Sequencing data consisted of 4 554 168 ITS-2 reads, with an average of 50 602 (s.d. 62 306) reads per sample. In total, 69 individual ASVs were identified, 65 of which were assigned to a single strongylid species: *Oesophagostomum aculeatum.* The remaining 4 ASVs were unidentified as strongylid taxa, assigned to superfamily trichostrongyloidea and could not be assigned at the genus level. The pairwise sequence distance (PSD) among all *Oesophagostomum* ASVs derived from the samples reached up to 10%, although such differences were restricted to 6 ASVs (8, 50, 51, 52, 57 and 67), with the PSD among all other ASVs not exceeding 5.4%. In the phylogenetic tree, all ASVs clustered within one strongly supported clade corresponding to *O. aculeatum*, with further internal structure (Fig. 1). One subclade comprised ASVs found in Bornean orangutans at both localities in Sabah, with 2 of these ASVs also detected in Japanese macaques in Jigokudani. Moreover, GenBank sequences originating from orangutans, in both Borneo (*P. pygmaeus*) and Sumatra (*P. abelii*), and Bornean white-bearded gibbons (*Hylobates albibarbis*) clustered within this subclade. The PSD within this subclade was below 2.5%.

Another small subclade comprised 5 ASVs (7, 9, 20, 23 and 30) detected in Japanese macaques across the 3 localities and one ASV (1) which occurred at all 3 Japanese localities as well as in crab-eating macaques at LKWS and orangutans at DVCA. This subclade also included 3 additional GenBank sequences from Japanese macaques. Other ASVs occurred across localities and host species, some occurred in multiple hosts at multiple localities, e.g. ASV 2 or ASV 22, some occurred in multiple hosts at a single locality, e.g. ASVs 25, 10 or 14, and some occurred in a single host at a single locality, e.g. ASVs 38, 27 or 69. Interestingly, these single-host-ASVs were always recorded in crab-eating macaques or Japanese macaques. It is worth mentioning that 2 sequences labelled as *O. bifurcum* in GenBank and originating from free-ranging bonobos (*Pan paniscus*) (Medkour *et al*., 2021) clustered in the *O. aculeatum* clade and differed by only 1.2–6.6% (below 3.7% for most haplotypes) from any *O. aculeatum* sequence.

Of the 4 ASVs assigned only to trichostrongyloidea, all were restricted to a single host species at a single locality, occurring only in crab-eating macaques at LKWS, with none showing a close identity match with any GenBank sequences. All 4 ASVs clustered together and formed a small subclade (SUP FIG 1) closely related to a clade comprising *Hyostrongylus* spp. and to a clade including *Ostertagia nianqingtanggulansis* (AJ577459, from unspecified host from Tibet), *Ostertagia* sp. (AB367797, Japanese Serow, Japan), *Mazamastrongylus dagestanica* (JQ925868, OM445254; moose, Russia) and *Spiculopteragia* spp. (European cervids). Although *M. dagestanica* is currently assigned to the family Trichostrongylidae, all other taxa are from the family Haemonchidae, suggesting the nature of the trichostrongylids detected in macaques.

**Figure 2.**
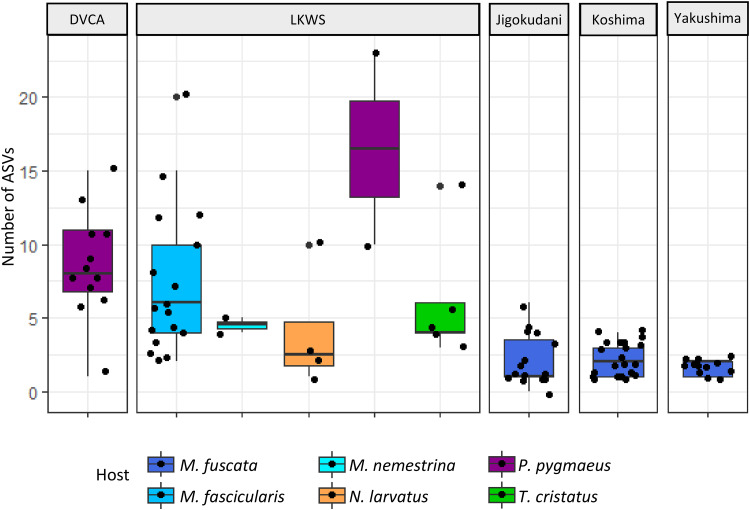
Amplicon sequence variant (ASV) diversity of strongylid nematodes detected in faecal samples of South-East and East Asian primates from 5 localities, indicated across the upper x-axis.

**Figure 3.**
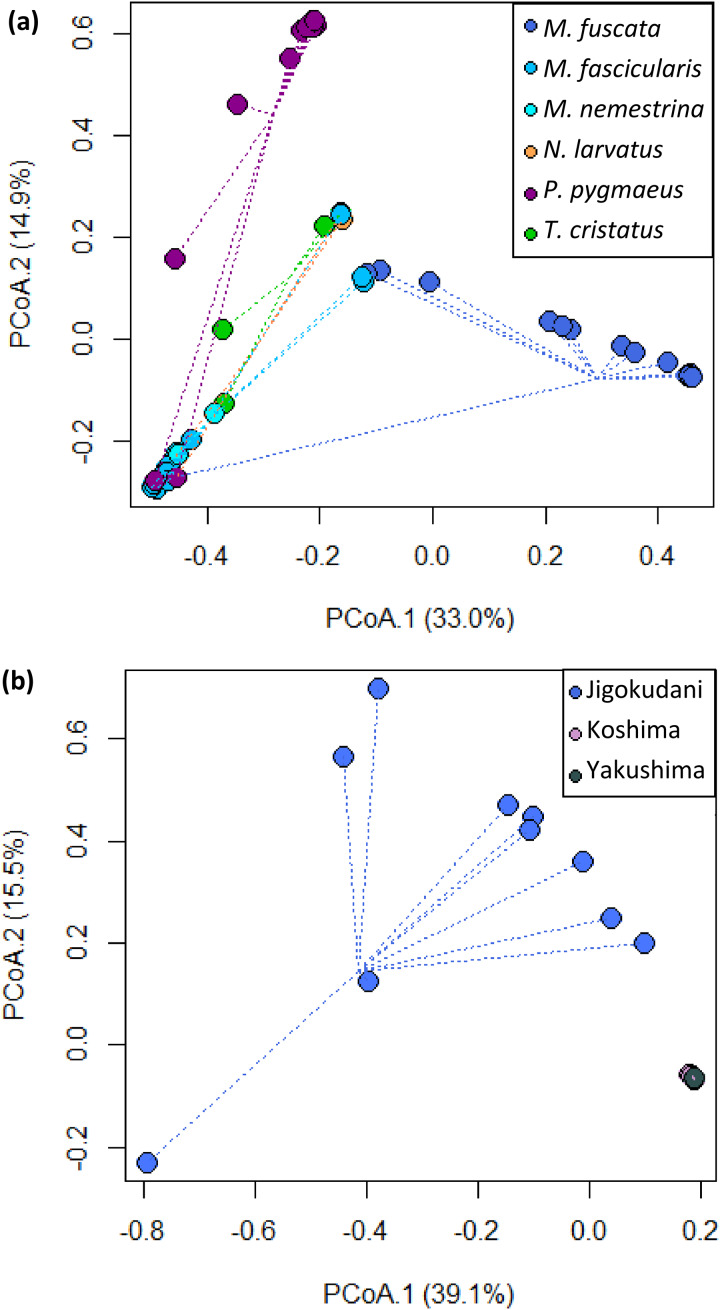
Principal coordinate analysis (PCoA) plots of the beta diversity (assessed through Bray–Curtis dissimilarities) among strongylid nematode communities of (a) 6 South-East and East Asian Primate species from 5 localities and (b) *M. fuscata* from 3 localities in Japan. Convergent dots indicate similarities in community composition. While Koshima and Yakushima are represented by multiple samples, close convergence of points shows no separation within ordination space at this scale.

Upon visual inspection, alpha diversity (at the level of individual ASVs) of strongylid communities appeared to show minimal variation between host species (Fig. 2). GLMMs found statistically significant differences in alpha diversity with *post hoc* analysis revealing differences between only Japanese macaques and other host species, with no alpha diversity differences found among sympatric host species at LKWS (*P* > 0.08 for all pairwise comparisons). Japanese macaques showed lower alpha diversity of strongylid communities compared to crab-eating macaques, silvery lutungs and orangutans (*P* < 0.001 for all pairwise comparisons), yet no differences were found between the 3 localities in Japan. However, ASV composition of strongylid communities showed significant interspecific variation, both between host species (ANOSIM: *R* = 0.597, *P* = 0.001) and amongst Japanese macaques in different localities (ANOSIM: *R* = 0.425, *P* = 0.001), supported by visual inspection of ordination plots (Fig. 3).

## Discussion

Faecal samples from 6 NHP species inhabiting regions of South-East and East Asia were examined to assess the diversity of their strongylid nematode communities. A community profiling approach was applied, based on a previously optimized and tested HTS amplicon sequencing methodology. Focusing on strongylid communities from multiple NHP species allowed the diversity of these communities to be investigated with reference to sympatric species, different localities and host phylogenies. The reported diversity of strongylid nematodes highlights the potential consistency of these communities in NHP hosts within South-East and East Asia. The limitations of HTS methods should be noted, most poignantly the restriction in taxonomic resolution due to short sequence lengths and potential for sequencing errors from PCR biases (Kircher and Kelso, 2010; Ambardar *et al*., 2016). However, previous work (Pafčo *et al*., 2018) has shown successful approaches for diminishing the impact of these stochastic effects.

There was a clear dominance of *O. aculeatum,* with nearly all ASVs assigned to this taxon. Dominance of *O. aculeatum* was expected, mirroring previous reports of strongylid infections in Asian NHPs (Frias *et al*., 2019). Still, the near complete absence of other strongylid taxa was surprising, as strongylid nematodes occur in complex communities comprised of multiple genera in other studied hosts. The majority of studied samples showed single species infections, contrasting with reports of strongylid infections in African NHPs (Pafčo *et al*., 2019; Mason *et al*., 2022; Ilík *et al*., 2023). Previously implemented molecular methods, involving individual larval specimens, identified the presence of a single larva of *Ternidens* in one Bornean orangutan (Frias *et al*., 2019), raising questions as to why this study did not identify the presence of *Ternidens*. The absence of *Necator americanus* among these samples should also be noted, as this strongylid has previously been reported in the local human population (Lim-Leroy and Chua, 2020) and is known to be frequently transmitted between humans and wild NHPs in Africa (Pafčo *et al*., 2019). Potentially, this study is restricted by the limited sample size of some host species (especially orangutans at LKWS), with further sampling required for a more complete picture of strongylid diversity among Asian NHPs.

ASVs of *O. aculeatum* found in this study were further separated into 2 clusters. The first cluster consisted mainly of ASVs from orangutans, with 2 additional ASVs identified from Japanese macaques. This cluster also includes 4 sequences from GenBank, recorded as infecting *P. pygmaeus*, *P. abelii* and *H. albibarbis*, suggesting a lineage specialized to orangutans, with historical spill-over to other hosts. This cluster assembled closely with the 2 sequences from bonobos registered as *O. bifurcum*, though based on clustering these sequences may in fact represent *O. aculeatum* (Medkour *et al*., 2021). The second cluster contained more ASVs and is identified in a larger diversity of host species, indicating it may be a more generalist lineage. Differentiation of these clusters as lineages or species would require morphological examination of adult worms and analysis of more markers. Cryptic species of *Oesophagostomum* have previously been proposed for nodular worm infections in humans and NHPs in Uganda (Ghai *et al*., 2014), with high genetic variability of *Oesophagostomum* spp. observed in African primates (Sirima *et al*., 2021; Mason *et al*., 2022). However, although the ITS-2 marker, also used in this study, is ideal for distinguishing between known species for which genetic data exists in repositories, it should be noted that this marker shows high levels of intraspecific variability, which may limit its value in identifying potential cryptic species (Conole *et al*., 1999; Poissant *et al*., 2021; Halvarsson and Tydén, 2023). Several host-specific lineages were previously found in *Oesophagostomum* following *cox1* analyses with 2 clear lineages showed within *O. aculeatum*, the first being found in a number of hosts, while the second being more host-specific (Frias *et al*., 2019). This confirms a certain degree of host preference within the *O. aculeatum* lineages detected and shows that involving genetic markers of divergence at the mitochondrial DNA loci would be an important aid in deciphering potentially cryptic species (Blouin, 2002).

The 4 trichostrongyloidea ASVs identified in crab-eating macaques highlight the current convolution in genetic distinction of strongylids in wildlife hosts. There are records of unspecified *Trichostrongylus* sp. based on the presence of eggs in various Asian macaque species (Kumar *et al*., 2018; Kurniawati *et al*., 2020; Fernando *et al*., 2022). Although humans and some African cercopithecoid primates are occasionally reported with *Trichostrongylus colubriformis* infection (Phosuk *et al*., 2013; Sharifdini *et al*., 2017; Obanda *et al*., 2019), and apparently there are *Trichostrongylus* spp. infecting African great apes (Mason *et al*., 2022), these sequences did not cluster with any *Trichostrongylus* spp. provided in the GenBank. Thus, as a more probable candidate *Nochtia nochti* is proposed, a trichostrongyloid nematode parasitizing *Macaca* spp. across Asia which unfortunately is not represented in available sequence databases. However, pathogenesis described in *N. nochti* (Yalcindag *et al*., 2021; Fonti *et al*., 2023) holds similarities to other trichostrongyloids from the family Haemonchidae (Deplazes *et al*., 2016), within which these sequences clustered.

The number of strongylid ASVs per sample, a quasi-measure of alpha diversity, was generally consistent across host species. Of all host species, only Japanese macaques showed noticeable differences (significantly lower number of ASVs) from other species. This finding may be as expected, with Japanese macaques being the unique sampled species occurring in East Asia, compared to the other species in South-East Asia. Of particular note is the variation identified between the 2 macaque species, Japanese macaques and crab-eating macaques, highlighting how geographical factors and sympatric species can influence strongylid communities of closely related host taxa. Japanese macaques are the world's most northern-living primate (Cozzolino *et al*., 1992; Tsuji *et al*., 2008), meaning it is one of the few NHP species that exists solely in single-primate communities (Ito *et al*., 2021). As such, the ability for parasites to host switch, a method whereby a parasite establishes within a novel host species (De Vienne *et al*., 2007; Cooper *et al*., 2012), is limited due to the absence of closely related species already hosting the parasite. Fossil evidence suggests that Japanese macaques may have long existed within a single-primate community (Marmi *et al*., 2004; Kawamoto *et al*., 2007), which may also be reflected among their intestinal parasite fauna (Gotoh, 2000).

Additionally, this northern distribution means Japanese macaques inhabit Japanese islands where environmental conditions differ quite dramatically from those in Borneo. Japanese macaques living at more northern latitudes, where the climate is characterized by colder winters, were previously shown to carry fewer gastrointestinal parasites at lower prevalence (Gotoh, 2000). The harsher seasonal environment in Japan may reduce the longevity of strongylids during developmental stages in the external environment (Knapp-Lawitzke *et al*., 2014). As such, ASV diversity, in Japan compared to Borneo, is limited by the robustness of strongylid taxa and their ability to withstand harsher environmental conditions.

Great variation between the samples according to the community composition of strongylids was observed both among species as well as within species (when concerning Japanese macaques). Some of this among-species variation is explained by the 2 identified lineages of *O. aculeatum*, with the divergent ordination of orangutans coinciding with the ‘*orangutan’* lineage, almost exclusively identified within this host. Likewise, the divergent ordination of crab-eating and pig-tailed macaques from Japanese macaques attests to the importance of sharing a habitat with other sympatric primates, supported by the widespread second lineage of ASVs identified within these hosts. Japanese macaques occupying a separate ordination space are additionally at least in part due to the reduced diversity of strongylid communities in this host species. This divergent ordination of macaque species, suggesting divergent composition of strongylid communities, contradicts the host–parasite co-speciation hypothesis (Brooks, 1979). Interestingly, only a single ASV was commonly identified among all 3 macaque species, with Japanese macaques sharing just 3 additional ASVs with either crab-eating or pig-tailed macaques, highlighting the role of environmental factors influencing parasite communities. Divergent ordination among Japanese macaques from different localities further emphasizes this, with hosts of the same species showing variation within strongylid community composition and only a single shared ASV identified across all 3 localities. Geographical isolation within Japanese macaques has led to the evolution of a subspecies in one locality: *M. f. yakui* in Yakushima (Hayaishi and Kawamoto, 2006). Host evolution is not mirrored by their strongylid communities in this case, however, with Yakushima strongylid communities showing convergence, in terms of composition, with Koshima, while Jigokudani strongylid communities show far greater divergence, again opposing the host–parasite co-speciation hypothesis. Further exploration of this hypothesis among NHPs, and environmentally driven deviation, could benefit from using the *Macaca* genus as a model, with 22 currently recognized species distributed into several lineages (Thierry, 2007) and occupying the widest geographical range of all NHP, spanning both tropical and temperate regions (Fooden, 1982).

Concluding, the strongylid nematode communities of South-East and East Asian NHP were dominated by *O. aculeatum*, with single-species occurrence identified in most samples, which is in sharp contrast to the rich strongylid communities harboured by African great apes and other African NHPs. The genetic distances between sequences suggests the potential for 2 cryptic lineages within *O. aculeatum*. One lineage appears more specialized in terms of host diversity, with the other being more generalist and diverse. However, ITS-2 does not provide a suitable marker for evaluation of intraspecific diversity, therefore further sequencing with the inclusion of more genetic markers is required to better decipher the genetic make-up of the *O. aculeatum* complex. These findings highlight how geographic variables may influence parasite communities within NHPs, disuniting host–parasite co-speciation. While the reported strongylid communities seem low in species richness, this report is only an initial insight and additional sampling is required to truly capture the genetic diversity of strongylid communities in Asian NHPs. Establishing baseline data on NHP strongylid infections can assist with ongoing monitoring of health threats to wild NHPs with HTS providing complex insights into strongylid community composition and ability to detect even less prevalent, but more pathogenic taxa.

## Supporting information

Mason et al. supplementary material 1 Mason et al. supplementary material

Mason et al. supplementary material 2 Mason et al. supplementary material

## Data Availability

Strongylid ITS-2 sequencing data is archived in the European Nucleotide Archive, under the accession number of the whole project PRJEB73875 (available at: https://www.ebi.ac.uk/ena/browser/view/PRJEB73875). Accession numbers for each sample are available alongside related metadata in Supplementary Table 1, which includes geographical location and sampling period.

## Supplementary material

The supplementary material for this article can be found at https://doi.org/10.1017/S0031182024000386.
